# Monarch butterfly population decline in North America: identifying the threatening processes

**DOI:** 10.1098/rsos.170760

**Published:** 2017-09-20

**Authors:** Wayne E. Thogmartin, Ruscena Wiederholt, Karen Oberhauser, Ryan G. Drum, Jay E. Diffendorfer, Sonia Altizer, Orley R. Taylor, John Pleasants, Darius Semmens, Brice Semmens, Richard Erickson, Kaitlin Libby, Laura Lopez-Hoffman

**Affiliations:** 1 US Geological Survey, Upper Midwest Environmental Sciences Center, La Crosse, WI 54603, USA; 2 Everglades Foundation, 18001 Old Cutler Road, Suite 625, Palmetto Bay, FL 33157, USA; 3 Department of Fisheries, Wildlife and Conservation Biology, University of Minnesota, St Paul, MN 55455, USA; 4 US Fish and Wildlife Service, Bloomington, MN 55437, USA; 5 US Geological Survey, Geosciences and Environmental Change Science Center, Denver, CO 80225, USA; 6 Odum School of Ecology, University of Georgia, Athens, GA 30602, USA; 7 Department of Ecology and Evolutionary Biology, University of Kansas, Lawrence, KS 66045, USA; 8 Department of Ecology, Evolution, and Organismal Biology, Iowa State University, Ames, IA 50011, USA; 9 Scripps Institution of Oceanography, University of California, San Diego, La Jolla, CA 92093, USA; 10 School of Natural Resources & the Environment, The University of Arizona, Tucson, AZ 85721, USA

**Keywords:** *Danaus plexippus*, extreme weather, forest loss, glyphosate, milkweed, neonicotinoid

## Abstract

The monarch butterfly (*Danaus plexippus*) population in North America has sharply declined over the last two decades. Despite rising concern over the monarch butterfly's status, no comprehensive study of the factors driving this decline has been conducted. Using partial least-squares regressions and time-series analysis, we investigated climatic and habitat-related factors influencing monarch population size from 1993 to 2014. Potential threats included climatic factors, habitat loss (milkweed and overwinter forest), disease and agricultural insecticide use (neonicotinoids). While climatic factors, principally breeding season temperature, were important determinants of annual variation in abundance, our results indicated strong negative relationships between population size and habitat loss variables, principally glyphosate use, but also weaker negative effects from the loss of overwinter forest and breeding season use of neonicotinoids. Further declines in population size because of glyphosate application are not expected. Thus, if remaining threats to habitat are mitigated we expect climate-induced stochastic variation of the eastern migratory population of monarch butterfly around a relatively stationary population size.

## 1. Introduction

The population size of migratory monarch butterflies (*Danaus plexippus*) in eastern North America has been highly variable over the last two decades but shows long-term declines based on annual monitoring of their overwintering colonies in central Mexico; the population declined 84% between the winters of 1996–1997 and 2014–2015 and faces a risk of quasi-extinction of 11–57% in the next 20 years [1]. A similar decline has been observed in the Western migratory population of monarch butterflies [2]. In response to declines in monarchs and other pollinators, on June 2014, the administration of President Obama issued a presidential memorandum calling for the restoration of pollinator and monarch habitat [3]. In August 2014, the US Fish and Wildlife Service (USFWS) was petitioned to list the monarch as a threatened species [4]. The decline in monarch numbers is particularly concerning because the species has a remarkably high societal and cultural value [5], and many educational [6], citizen science [7] and conservation [8] programmes engage people with monarchs. To effectively conserve this imperiled species, understanding the threats affecting annual abundance is important [9]. Here, we use partial least-squares regression and time-series analysis to investigate potential factors influencing monarch overwintering population size from 1993 to 2014.

Monarchs face a variety of threats potentially affecting their population size in Mexico. These threats may operate at different times of the life cycle, affecting survival in winter, breeding and survival in summer, and the success of migration between. A leading hypothesis for the decline of the eastern monarch population is habitat loss across its range [10–15]. Habitat loss in the overwintering sites in Mexico is primarily caused by climatic trends and illegal logging, although rates of logging have slowed dramatically in recent years [11,13]. Milkweeds (mostly in the genus *Asclepias*) are essential host plants for monarch larvae; therefore, loss of milkweed and nectar resources in the breeding grounds of the Upper Midwestern US is another risk [12]. For example, milkweed abundance in Iowa declined 58% from 1999 to 2010, due primarily to herbicide usage associated with increased planting of genetically modified glyphosate-resistant corn and soybeans [12]. Agricultural conversion of habitat associated with Conservation Reserve Program (CRP) lands also poses an ongoing threat [16]. Due primarily to the loss of milkweed, monarch reproduction in the Midwest was estimated to have been reduced by 81% over this time period [1].

Monarch population dynamics are influenced by climatic factors, including temperature and precipitation during the overwintering, migration and breeding seasons [17–24]. Increasing climate variability, extreme weather events and climate change may pose a threat for monarchs [17,19–21,25,26]. For example, predictions of northern range shifts of monarchs and their ecological niche during the breeding season could lead to longer migration times and potentially reduced survival, or alternatively, reduced range size if monarchs are unable to track changing conditions [19,20]. Winter storms have caused high levels of mortality in overwintering colonies [25], and climate change may result in extensive portions of overwintering habitat becoming unsuitable for monarchs [17] and oyamel fir (*Abies religiosa*) trees (which harbour wintering monarchs) [26] within the next 40 years.

In addition to habitat loss and climate and weather factors, monarchs face a variety of other well-documented threats. For example, the protozoan parasite *Ophryocystis elektroscirrha* (*OE*) can have high rates of infection in monarch populations and can reduce survival, mass, flight speed, flight endurance and lifespan [10,27,28]. Insecticide use is also of concern for monarch populations; recently, the use of neonicotinoid insecticides has been implicated in delayed development times and smaller body sizes in monarchs, and elevated mortality rates, reduced population persistence, behavioural changes and slower development times in other pollinators [29–31]. Additionally, insecticides commonly used for mosquito control kill monarch larvae and adults [32,33].

Existing models of monarch population dynamics have not comprehensively examined the role of multiple factors driving the observed population decline. Previous investigations of monarch population declines and threats to monarchs have focused on documenting population trends and extinction risk [1,12,34], assessing potential impacts of climate on breeding [19–21,24], threats to the overwintering habitat [17,35–37], the relationship between monarch population size and breeding habitat loss [10,38], the effects of the loss of agricultural milkweed on monarch fecundity [39,40], and understanding demographic relationships across generations within the migratory cycle [14,34,41,42].

We used partial least-squares regression models and time-series analysis to examine the influence of multiple factors on monarch butterfly overwintering population size from 1993 to 2014 (table 1). Our list of potential factors was informed by the published literature [10,13,15–31,37]. We examined potential threats to the monarch population for which large-scale, range-wide data existed; this precluded investigating some potential threats that had inadequate time-series data such as nectar availability in breeding and migration.

**Table 1.** Description of variables, with variable names, related to annual estimates of eastern monarch butterfly population size. Number of years in which missing data were interpolated is provided per variable. Citations supporting the covariate are provided. Period 1: 1–10 May; Period 2: 11–20 May; Period 3: 21–30 May; Period 4: 31 May–9 June.

variable categories	variable names	definition	citation	missing
density dependence	apva_1yr	population size in the previous year	[43]	1
survival	closum, dinsum, thisum, imisum	total regional neonicotinoid (by chemical) applied 1994–2009 (kg)	[26,30,44]	2
	totalneon	total regional neonicotinoids (sum of all chemicals) applied (kg)	[26,30,44]	2
	Prop_Inf	proportion of larvae infected with protozoan parasite *Ophryocystis elektroscirrha* (*OE*)	[10,27,28]; S. Altizer, unpublished data	7
	LDD	regional number of days exceeding lethal maximum temperature threshold	[45]; L. Ries, pers. comm.	0
	NighttempF	for South, mean night-time temperature in the autumn (1 Sept–30 Nov)	[46]; O.R. Taylor, unpublished data	0
	total precipitation	total precipitation for the overwintering location (13 Dec–31 Dec)	[15,21,22]	0
	mean, minimum and maximum wind speed and maximum wind gust	wind conditions for the overwintering location (13 Dec–31 Dec)	[15,21,22]	0
	mean, minimum and maximum temperature and humidity	weather conditions for the overwintering location (13 Dec–31 Dec)	[15,21,22]	0
reproduction	Prec	for South, total annual precipitation	[16,19,20,22,47]	1
	MeantempSp	for South, mean temperature in the spring (1 Mar–30 Apr)	[16,19,20,47]	1
	T70p(1–4)sum	days greater than 21.1°C (greater than 70°F) in Periods 1–4	[16–20]; O.R. Taylor, unpublished data	1
	Tempp(1–4)avg	temperature average in Periods 1–4	[16–20]; O.R. Taylor, unpublished data	1
	drought	standardized precipitation index (1 June–30 Aug)	[16–20,48]	0
	MeanJ	mean temperature in June	[16–20]	0
	TminJu	percentage of days less than 10th percentile for minimum temperatures in June	[16–20]	1
	TminJl	percentage of days less than 10th percentile for minimum temperatures in July	[16–20]	1
	TminAug	percentage of days less than 10th percentile for minimum temperatures in Aug	[16–20]	1
	GDD	regional number of days within the suitable threshold for growth	[39,47,49]	0
habitat availability	glysum	total regional glyphosate applied 1993 to 2009 (kg); imputed greater than 2009	[12,15,50,51]	2
	glycum	cumulative regional glyphosate applied 1993 to 2009 (kg); imputed greater than 2009	[12,15,50,51]	2
	DC	regional sum of dicamba applied (kg)	expert opinion	2
	TwoD	regional sum of 2,4-D applied (kg)	expert opinion	2
	CRPsum	available CRP in the region (ha)	[40,50,52]	0
	Ramirez.cumul and Vidal.cumul	cumulative loss of overwintering forest in central Mexico (ha)	[11,13,26]	2 and 1

## 2. Material and methods

We examined the role of multiple factors on monarch butterfly overwintering population sizes observed annually in Mexico from 1993 to 2014 [44]. Population size data were based on the amount (ha) of overwintering habitat occupied by eastern migratory monarchs when they congregate in the high-elevation oyamel fir forests of central Mexico. Since the vast majority of the eastern migratory population of monarchs alive at the end of each year is in these colonies, the area that they occupy is a proxy for the total population size (assuming relatively constant density in their overwintering sites [53]). We used estimates of monarch butterfly overwintering population size from a population viability analysis [1], a Bayesian state-space model analysis that produced estimates of the population size from 1993 to 2014 while mathematically controlling for observation error. We assumed this time series of overwinter abundance was generated by a general process:

2.1 dx=f(x, θ)dt+g(x, θ)dW,

where *x* is the population state, *f*(*x, θ*) describes deterministic aspects of the time series, and *g*(*x, θ*)d*W* determines stochastic aspects of the system.

We included a list of potential threats and climatic factors informing these deterministic and stochastic components from a review of the published literature and expert opinion elicited at the ‘Continental scale monarch conservation planning’ working group meeting at the US Geological Survey's John Wesley Powell Center for Analysis and Synthesis and further refined by participants of the US Monarch Conservation Science Partnership. For each of three regions, we examined more than two dozen principal factors considered important in monarch population dynamics for which large-scale, range-wide data existed (table 1).

**Figure 1.**
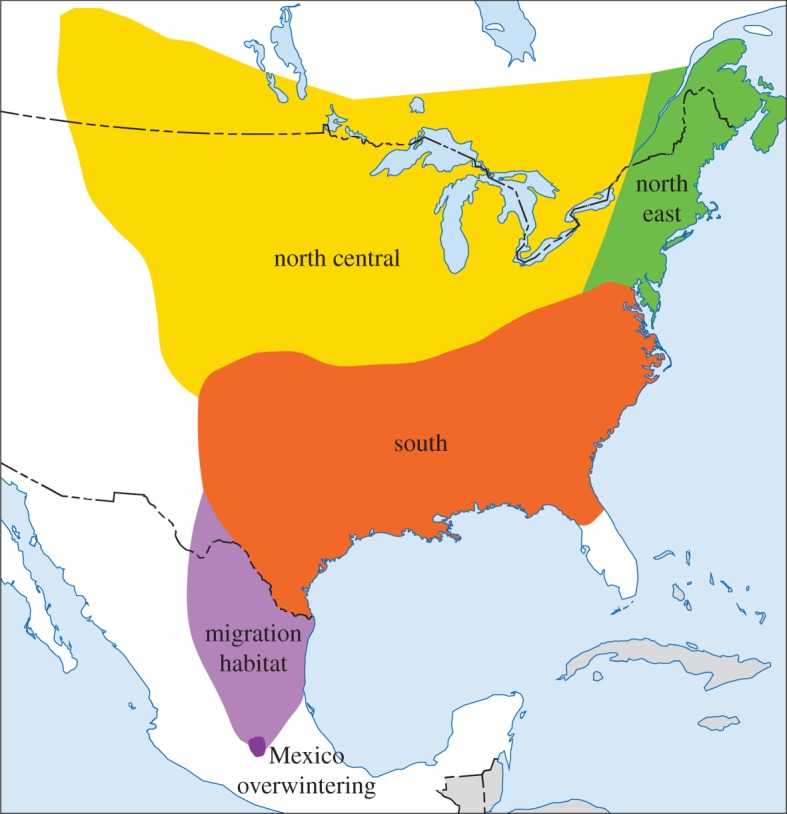
Range of the eastern migratory population of monarch butterfly. Southern, north central, and northeastern regions are occupied during breeding season.

We segregated the monarch life cycle into regions based on Oberhauser *et al*. [14] (figure 1). Our model examined factors across the breeding region that included the southern USA, and the north central and northeast breeding areas; for non-climatic factors, the north central and northeast were summed into a northern breeding region. Unfortunately, many of the data sources we gathered do not have ready-made counterparts for southern Canada; we assumed data from these regions correlated with those of their geographic counterpart in the USA. This assumption seems reasonable given that the vast majority of northern-breeding butterflies breed in the American portion. We also examined climate and habitat loss occurring in the Mexican overwintering area.

### 2.1. Habitat availability

We estimated habitat loss factors by breeding region including the southern USA and the North—which included both the north central and the northeast breeding areas (figure 1). We summed the north central and northeast as they encompass similar stages (generation numbers 2, 3 and 4) in the monarch's annual life cycle. Habitat loss included several proxies of milkweed and nectar resource losses, including herbicide use (dicamba, 2,4-D, and glyphosate, which reduce milkweed densities in agricultural fields [12,15]), and extent of beneficial habitat, Conservation Reserve Program (CRP) land [52], which has declined in the last decade [16,52]. Our model included both annual glyphosate (*N*-(phosphonomethyl)glycine) use from 1993 to 2014, and cumulative glyphosate use per region over time [50]. Because agricultural fields were important sources of milkweed and monarchs in 2000 before widespread adoption of glyphosate-resistant crops [54], this value is a proxy for monarch habitat lost [55]. Total kilograms from 1993 to 2014 of two other commonly used herbicides were also included (2,4-dichlorophenoxyacetic acid (hereafter 2,4-D) and 3,6-dichloro-2-methoxybenzoic acid (hereafter dicamba)), obtained from the US Geological Survey's Pesticide National Synthesis Project [50]. 2,4-D is one of the oldest, most widely used herbicides in the world, used commonly on lawns, cereal crops, pastures and orchards. Dicamba, on the other hand, is often used in pastures, fence-rows and roadsides to control brush, bracken and broadleaf weeds. Lastly, we used the total number of hectares of CRP land from 1993 to 2014 [52].

We examined annual forest loss in the overwintering area [13,26] made available through use of high-resolution aerial photography, multi-resolution satellite imagery, and field surveys. Both studies reported loss over several-year periods, which we annualized to create an annual time series. For the sake of our analyses, we assumed for the Vidal *et al.* [13] time series that there was no forest loss prior to 2001; because one study ended in 2012 [26] and the other in 2013 [13], we used interpolative methods to predict the missing years to 2014 (see Missing data). These studies are similar in that they demonstrate cumulative forest loss, but they differ in the reported total amount of forest loss; therefore, their separate use is a matter of parametric uncertainty.

### 2.2. Reproduction

We obtained monthly total precipitation and temperature maxima and minima data for the conterminous USA for each month between 1993 and 2014 from the PRISM dataset (4 km resolution, PRISM Climate Group, Oregon State University, http://prism.oregonstate.edu) [48]. We estimated climate factors for the southern, north central and northeast US breeding areas (figure 1) that our expert team hypothesized were important for monarch migration and fecundity, or that had been identified in previous studies [22–24]. Total precipitation and mean temperatures in the South from 1 March to 30 April were included from 1993 to 2013. Drought intensity from 1993 to 2014 (as determined by the Standardized Precipitation Index, which measures the number of standard deviations that cumulative precipitation deviates positively or negatively from the mean [46] was used in the South (for the period 1 March to 31 May) and in the north central and northeast (1 June to 30 August) to coincide with the presence of monarchs in these regions. For the north central and northeast, we also included mean temperatures and total number of days greater than 21.1°C (greater than 70°F) for each 10-day period between 1 May and 10 June from 1993 to 2014, mean temperatures in June from 1993 to 2014**,** and monthly extreme cold events in June to August (measured as percentage of days below the 10 percentile for minimum temperatures) from 1993 to 2012. In addition, we included mean night-time temperatures [46] from 1 September to 30 November for the South in autumn, from 1993 to 2014. Each of these factors are expected to influence reproduction through reduction in host plant quality, delayed growth of larvae and potential mismatching of host plant availability and monarch presence.

We included growing degree days (GDD) for monarchs from 1993 to 2014, which accumulate the degrees that can contribute to development within a suitable temperature range (11.5°C to 36°C) [49,56]. An average of 352 GDD is needed for an egg to develop into an adult monarch [39,49]; daily GDD are calculated using the mean of the day's highest (less than 36°C) and lowest temperatures. The minimum temperature required for growth (11.5°C) was subtracted from the mean value to estimate the daily GDD value. We calculated GDD in the South from 22 March to 13 June for spring, and from 6 September to 21 November for autumn. For the north central and northeast region, we calculated GDD from 3 May to 12 September. Regional GDD estimates are based on mean accumulated GDD from a number of locations within each region (51 sites in the South, 54 sites in the north central and 35 sites in the northeast).

### 2.3. Survival

We included the proportion of the eastern population of monarch butterfly infected with the protozoan parasite *OE* from 1993 to 2012 in the overwintering and the autumn migratory population (data provided by S. Altizer, Project Monarch Health). Infection by *OE* leads to reduced survival [27]; as such, infected monarchs are less likely to complete migration [10,28]. We imputed the two years of missing values.

We also used total kilograms per hectare of several kinds of neonicotinoid insecticides used in the southern USA, and the north central and the northeast breeding areas [57]. For neonicotinoids, clothianidin was estimated from 2004 to 2008, dinotefuran was estimated from 2005 to 2009, imidacloprid was estimated from 1994 to 2009, and thiamethoxam was estimated from 2000 to 2009; these four chemicals were the most commonly used neonicotinoids during the period of study. Before the years 2004, 2005, 1994 and 2000, clothianidin, dinotefuran, imidacloprid and thiamethoxam, respectively, were not widely used. We also measured total neonicotinoid use per region by summing the four different varieties. We recognize the potential for delays in the effect of agricultural chemicals and their ability to persist in the environment [29,58–60] but we dispensed with lagged effects for the sake of parsimony. Because most mosquito control efforts occur in places with high human densities and most models suggest that rural areas are more important to monarch production [32], we did not consider insecticides mainly used to control mosquitoes and instead focused on those used to control agricultural pests.

Similar to growing degree days, lethal degree days (LDD), which count the degrees that are lethal or have sublethal fitness effects for monarchs (≥38°C [45]), were assessed from 1993 to 2014 for the breeding regions. The highest daily temperature was recorded for days reaching the threshold of 38°C and then 37°C was subtracted.

We examined weather-related effects during the overwintering period with mean, mean minimum, and mean maximum temperature (°C), humidity (%), and wind speed, maximum wind gust (m s^−1^), and total precipitation (mm) for each year for 13–31 December, when monarch population size is assessed by World Wildlife Fund-Mexico and their partners. Because there are considerable gaps (particularly in the mid-1990s) in the temperature and precipitation data for the weather station nearest to the Monarch Butterfly Biosphere Reserve (Toluca, Mexico), data were obtained from a numerical meso-scale model [61].

### 2.4. Statistical analysis

#### 2.4.1. Missing data

We used a data interpolating empirical orthogonal function [62,63] to interpolate missing data with the *sinkr* package [64] in R [65]. The *sinkr* package interpolates missing values by decomposing the dataset via singular value decomposition (factorization of the data matrix into two orthogonal matrices and a diagonal matrix) until an optimal solution is found compared with a set of reference values (i.e. existing data). After providing initial guesses to the missing data, each subsequent iteration involves treating some of the known data as missing, inferring values for these known but treated-as-missing data, and then calculating the root-mean-square error between the known and inferred value. When the root-mean-square error is minimized, the interpolations for the missing data have been optimally identified relative to the characteristics of the known data.

#### 2.4.2. Variable reduction

Because there are many different factors potentially affecting long-term and annual changes in monarch butterfly population size, and relatively few years of measured overwintering population data, application of linear regression is problematic due to non-independence of environmental covariates, lack of statistical power and the associated inability to differentiate effects of covariates without risk of overfitting models [66]. To tackle this problem, we employed a data reduction technique, partial least-squares regression [66–68] on our full dataset of variables (table 1), to reduce the dimensionality of the covariate information and address the concern for multi-collinearity. Unlike principal components analysis and other similar dimension reduction procedures, this approach is ‘y-aware’, extracting latent components from the predictor variables that maximize covariance with the response (monarch population size) [66]. Partial least-squares analyses have three simultaneous objectives: the best explanation of the **X**-space (the set of environmental predictors), the best explanation of the **Y**-space (the biological response) and, importantly, the greatest relationship between the **X**- and **Y**-space. We used this y-aware dimension reduction to identify a subset of variables for use in subsequent time-series analyses of annual population size. This shortened list consisted of the variables with the greatest absolute loading (i.e. the largest correlation) on each component from the partial least-squares regression results (loadings ≥ 0.15).

To determine model performance of these partial least-squares regressions, we calculated the fitted *R*^2^. Additionally, we conducted a complete leave-one-out cross-validation and, for each model, calculated the predicted residual error sum of squares (PRESS) and *Q*^2^, also known as the cross-validated *R*^2^ [69]. The PRESS statistic is calculated as $\text{PRESS}=\Sigma_{i=1}^{n}{\left(y_{i}-{\hat{y}}_{i/i}\right)}^{2}$, where the notation *i*/*i* indicates that the response is predicted by a model estimated when the *i*-th sample was left out from the training set. The cross-validated *R*^2^ or *Q*^2^ is calculated as: $R_{\mathrm{CV}}^{2}\equiv Q^{2}=1-(\text{PRESS/TSS})=1-(\Sigma_{i=1}^{n}{\left(y_{i}-{\hat{y}}_{i/i}\right)}^{2}/\Sigma_{i=1}^{n}{\left(y_{i}-\bar{y}\right)}^{2})$ (where TSS is the total sum of squares). Analyses were conducted in R [65] with the *plsdepot* package [70].

#### 2.4.3. Time-series analysis

We selected the most highly loaded variables (loadings >|0.15|) from the partial least-squares regression components (i.e. 10 variables from each component). We further narrowed the set of variables by selecting among the variable set those variables with *r* > 0.6. For instance, when variables for multiple regions expressed high loadings and were highly correlated, we selected the variable for the north central region because of its importance to breeding [14]. We selected the overwinter forest loss variable with the higher loading. Using this reduced set of 11 variables, we then calculated time-series regressions [71,72]. Annual overwinter population size was estimated with an autoregressive model [1] implying a density-dependent relationship [43] between abundances in year *t* and *t* + 1. Thus, we modelled the time series of estimated population size as a Gompertz model for log index with environmental factors as covariates [47,73], with the general form:

2.2 $$N_{t}=\alpha_{0}+\beta (X_{t})+\varepsilon_{t},$$

where *N_t_* is the natural log of population size in year *t*; *α*_0_ is a constant representing the intrinsic rate of population growth; **X** is the design matrix including explanatory variables relating to abundance in the previous year (*N*_*t*−1_), habitat loss, parasite infection, insecticide exposure, and climate and weather factors on population size in year *t*; *β* quantifies the effects of these variables on *N*_*t*_; and *ε_t_* is the random error component representing unknown environmental variation and is normally distributed (white noise) with zero mean and variance *σ*^2^.

All time-series models were limited to ≤4 predictor variables due to the limited sample size of the response variable. This combination of 11 predictor variables taken 4 at a time led to a model set of 330 models. Models were ranked according to their Bayesian information criterion (BIC), variable importance was calculated based upon the sum of the model weights, and inferences were made from the best-supported models (≤10 BIC units of the highest-ranking model). Model-averaged parameter estimates were calculated from the best-subset of covariates with estimates shrunk toward 0 in accordance with variable importance [74]. Models were assessed for residual temporal structure by plotting the autocorrelation function and compared with BIC to general least-squares regression alternatives.

Lastly, the best-subset regressions suggested that glyphosate application was the most commonly associated covariate with overwinter population size. A number of recent studies [12,15,22,38,51] link the loss of milkweeds, the sole host plant of monarch butterflies, in Midwest corn and soybean fields to the use of glyphosate. We used estimates of milkweed resource in the north central US provided by Pleasants [51] in a structural equation model examining a cause–effect relationship among these variables, written as:

$$\begin{array}{rl} & \text{log(overwinter population size)}=\delta \cdot \text{log(milkweed resource)}\\ & \text{log(milkweed resource)}=\gamma \cdot \text{log(cumulative glyphosate application)}. \end{array}$$

This structural equation model was calculated with the *lavaan* package [75] in R; model fit was assessed with the root-mean-square error of approximation index (RMSEAI), and the comparative fit index (CFI). We used R v. 3.3.2 for all statistical analyses; our model code is available in the electronic supplementary material, appendix B.

## 3. Results

**Figure 2.**
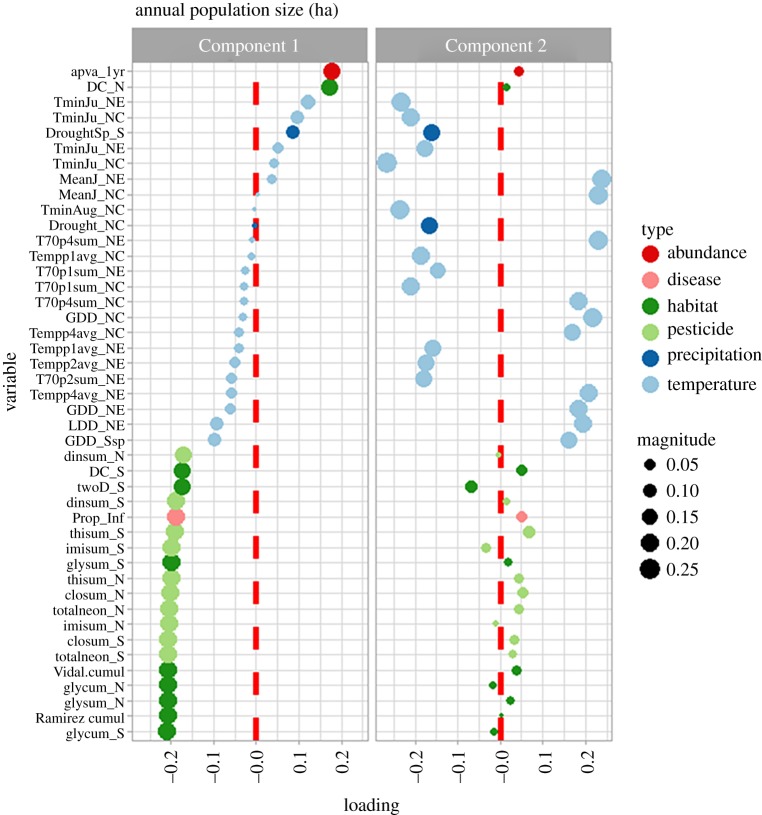
Potential threats affecting the eastern migratory population of monarch butterfly across the annual cycle, as described by a partial least-squares regression. Component 1 is the first component and Component 2 is the second component of the partial least-squares regression. Variable names are provided in table 1. To increase ease of display, only variables with loadings > |0.15| on at least one of the components are shown; circle size depicts relative magnitude of loading.

Partial least-squares regression indicated that two components explained 91.1% of the variation in overwinter population size. The *Q*^2^, or cross-validated *R*^2^, was 86.6%, suggestive of good predictive ability. The first component explained nearly six times the variation in overwinter population size compared with the second component (77.8% versus 13.3%). This first component loaded heavily on slowly evolving covariates such as herbicide and insecticide application rates, population size in the previous year and infection by *OE* (figure 2). The second component loaded on stochastically varying variables related to climate, principally measures of temperature during June.

The direction and strength of the variable loadings indicated population size in the previous year was a positively associated determinant of population size, as was total extent of Conservation Reserve Program land in the North and South. Glyphosate use and neonicotinoid application in all regions, and parasitism by *OE*, were negatively associated with population size. Glyphosate use, followed by the amount of forest loss in the overwintering area and neonicotinoid use in the breeding period, had the strongest negative loadings (or correlations with population size). Counterintuitively, dicamba and 2,4-D application in the northern regions was positively associated with population size (both herbicides declined in use in the North at the time that monarchs were also in decline); however, in the South, the use of these herbicides was negatively associated with population size (both herbicides increased in use in the South over the period of study).

Stochastically varying climatic factors largely expressed strong loadings on the second component (figure 2). Phenological patterns in climate appeared to influence population size; in general, earlier warm temperatures (mean temperatures and number of days greater than 21.1°C from 1 to 20 May) were negatively associated with population size, whereas later warm temperatures (31 May–9 June) in the northern regions were positively associated. As expected, growing degree days were positively associated with population size, as were warmer temperatures in the month of June in the north central and northeast region. Curiously, population size declined as the minimum temperature in summer months increased in the north central regions. Drought in all regions (as characterized by the second component) had a deleterious effect on population size.

Correlation among covariates shared across regions was generally high for the slowly evolving covariates (e.g. cumulative glyphosate application in the North and South was correlated *r* = 0.999). Thus, regression models were constructed with covariates from a single region, the north central. The best subset of reduced-variable models, comprising a cumulative sum of model weights equal to 0.94 and none with more than three covariates, comprised nine models (table 2). Eight of nine models included cumulative glyphosate application; the top model also included the number of days in Period 1 with temperature greater than 21.1°C (greater than 70°F) and minimum temperature in August. These three variables each had variable importance measures of greater than 0.91; the other variables in the best subset had variable importance measures less than 0.04. None of the best subset of models included dicamba or total neonicotinoid application. Further, each of the models but one in the best subset possessed a slowly evolving covariate (e.g. glyphosate, overwinter forest loss) explaining trend, obviating need for the previous year's abundance or an autoregressive correlation structure.

**Table 2.** Standardized coefficients and model parameter estimates for the best subset of models fitted to eastern migratory monarch butterfly overwinter population sizes for 1993–2014. Variable importance and average model coefficients are provided. Variable acronyms are described in table 1.

glyphosate^a^	Tmin Aug^b^	T70p1 sum^c^	Tmin Jun^d^	T70p4 sum^c^	OW forest^e^	mean JuneT^d^	GDD^f^	previous year abundance	*R*^2^	logLik	BIC^g^	ΔBIC^h^	*ω*^i^
−0.506	−0.131	−0.139							0.929	10.86	−6.26	0.00	0.919
−0.530		−0.118	−0.091						0.897	6.67	2.12	8.38	0.014
−0.513		−0.160					0.079		0.896	6.58	2.30	8.56	0.013
−0.517	−0.124			0.093					0.895	6.51	2.44	8.70	0.012
−0.519	−0.130					0.091			0.895	6.48	2.49	8.75	0.012
−0.504		−0.154							0.877	4.73	2.91	9.17	0.009
	−0.165				−0.338			0.213	0.890	6.03	3.40	9.65	0.007
−0.536	−0.109		−0.093						0.890	6.03	3.40	9.66	0.007
−0.337	−0.155				−0.192				0.890	5.96	3.53	9.79	0.007
−0.501	−0.122	−0.129	−0.003	0.002	−0.006	0.002	0.001	0.002	averaged model coefficients (shrunk)				
0.990	0.926	0.912	0.031	0.025	0.021	0.020	0.015	0.013	variable importance				

^a^Cumulative glyphosate application in the northern USA.

^b^Minimum temperatures in August in the north central USA.

^c^Number of days with temperatures greater than 21.1°C (greater than 70°F) in the north central USA, Period 1 and 4, respectively.

^d^Minimum and mean temperatures in June in the north central USA.

^e^Cumulative loss of overwintering forest.

^f^Growing degree days.

^g^BIC is the Bayesian information criterion.

^h^ΔBIC is the difference between the best model and the model of interest.

^i^*ω* is the model weight, calculated as $\omega_{i}=\text{exp(}-(1\text{/}2)\Delta_{i}\text{) /}\Sigma_{i=1}^{R}\text{exp(}-(1\text{/}2)\Delta_{i}\text{) }$, for R models.

**Figure 3.**
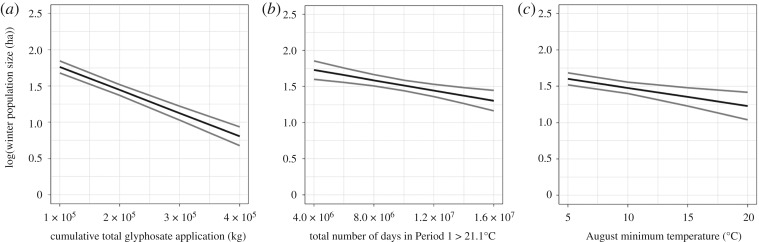
Principal environmental correlates of the monarch butterfly. The natural log of monarch butterfly population size (ha) predicted by major predictor variables in the top best subset regression models. Grey bands display 95% confidence intervals.

**Figure 4.**
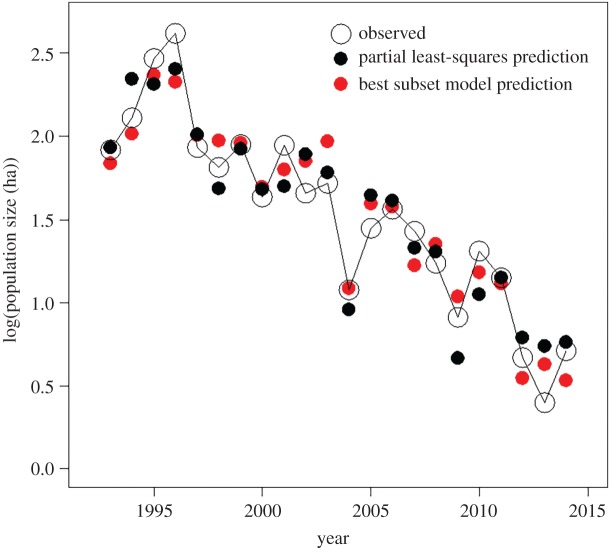
Annual overwinter population size of the eastern migratory population of the monarch butterfly predicted by partial least-squares regression and a reduced-variable linear regression, compared to observed population size.

The best time-series regression model, explaining 93% of the variation in overwinter population size, had a model weight of 0.919, nearly six times the weight of all other models combined. Cumulative glyphosate application was strongly negatively associated with overwinter population size (figure 3*a*; electronic supplementary material, appendix C). Both the number of days with temperature greater than 21.1°C (figure 3*b*) and minimum temperature in August (figure 3*c*) were moderately negatively associated with overwinter population size. The best reduced-variable model explained patterns in overwinter population size (*r* = 0.96) as well as the partial least-squares regression (*r* = 0.95) (figure 4).

**Figure 5.**
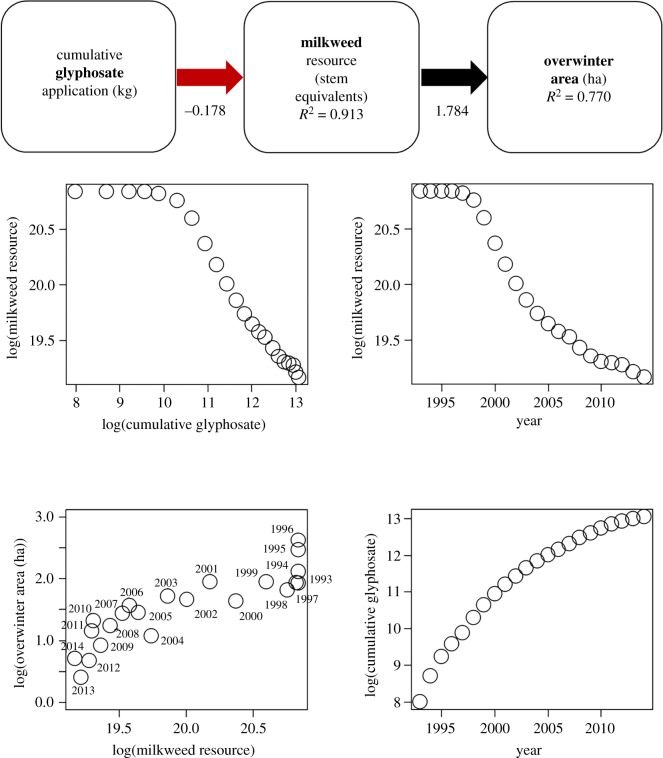
Structural equation model and plots exploring the relationships among butterfly population size, carrying capacity of the breeding area in terms of milkweed resource and glyphosate application. The path diagram describes overwinter area of eastern migratory monarch butterfly population as a function of the amount of milkweed in the northern USA, which in turn is a function of the cumulative application of glyphosate. The red arrow describes a negative association, whereas the black arrow describes a positive association; the magnitude of the parameter estimate is provided below.

The structural equation model fit the data well ($\chi_{1}^{2}=0.019,$ *p* = 0.89; RMSEAI less than 0.0001, CFI = 1.00), supporting a negative causal relationship of glyphosate application on milkweed resource (standardized *γ* = −0.178, s.e. = 0.033, *p* < 0.0001, *R*^2^ = 0.913), and a strong positive causal relationship of milkweed resource on overwinter population size (*δ* = 1.784, s.e. = 0.229, *p* < 0.0001, *R*^2^ = 0.770) (figure 5).

## 4. Discussion

A better understanding of major drivers of monarch population dynamics can inform the multitude of ongoing conservation efforts invested in this species. We found that the long-term drivers of population size in the eastern migratory population of monarch butterflies are slowly evolving factors relating to changes in habitat amount and quality. Annual stochastic changes in population size principally reflect changes in climatic conditions early in the northern breeding period [47].

Strong partial least-squares regression loadings on the first component implicated a number of factors potentially responsible for the long-term decline in overwinter population size. The principal variables were glyphosate and neonicotinoid use in the breeding period and total forest loss in the overwintering area. Neonicotinoid exposure results in increased development times, higher mortality rates and smaller body weights for monarchs [30] and has been associated with the decline of Lepidoptera in the UK and California [76,77]. Total neonicotinoid usage increased 48-fold in the northern USA between 2003 and 2010 (electronic supplementary material, appendix A), but thereafter began declining, probably lessening the correlation between neonicotinoid application and monarch butterfly abundance. Total forest loss in the overwintering area was a strong correlate of monarch butterfly abundance, irrespective of the data source considered [13,26]. The strongest loading, however, and the covariate identified in eight of the nine top performing reduced-variable linear models suggested that glyphosate application, by removing their obligate host plant, was the stressor most highly associated with the decline of the eastern migratory population of monarch butterflies.

After controlling for the long-term decline in monarch butterfly abundance, we found, similar to other studies, that annual monarch population dynamics were strongly influenced by climatic factors [17–20,24]. Strong loadings by minimum and mean June temperature, as well as temperature in Period 4 (31 May–9 June), on the second component of the partial least-squares regression indicated that temperature variation in the early growing season had an important influence on subsequent overwinter population size. Earlier warm temperatures (mean temperatures and number of days greater than 21.1°C from 1 to 20 May) in the northern regions were negatively associated with population size, whereas later warm temperatures (31 May–9 June) were positively associated with abundance. As minimum June temperature increased, however, population size declined. These results cohere with Zipkin *et al*. [24, p. 3045], who reported that the accumulation of monarch growing degree days had the strongest positive impact on counts of monarch butterflies in Ohio in the coolest sites, but that this effect diminished as sites became warmer, with the pattern beginning to reverse at the warmest sites. These temperature-abundance correlates suggest the possibility of a phenological mismatch, either between monarchs and their host plants or nectar sources, and diminished access to optimal resources [24] in early warm years. Minimum temperature in August was negatively related to abundance, perhaps because heightened temperatures forestall reproductive diapause and the subsequent onset of migration. Unlike Zipkin *et al*. [24], we did not find strong effects of precipitation on annual patterns in abundance, perhaps because precipitation, unlike temperature, exhibits considerably more spatial and temporal heterogeneity, which may have been dampened at the regional scales we examined.

Ecological processes are often extremely complex and, as a result, it can be difficult to discern putative cause-and-effect relations when there are many more predictor variables than there are samples. Small sample sizes preclude complex model development using traditional regression techniques and many environmental covariates associated with biotic responses often covary. The partial least-squares regression approach we took simultaneously maximizes the explained variation in the *X*-space (predictor variables), the explained variation in the *Y*-space (response variable), and the conjoint variation of the *X*- and *Y*-spaces (predictor and response variables). Despite the strengths of the partial least-squares approach, there are still uncertainties remaining that we cannot overcome with the data at hand. For instance, our ability to predict change in population size from one year to the next is hampered by noisy, collinear variables. Glyphosate application (irrespective of whether measured as cumulative or annual) obviated the need for previous year's abundance, derogating the role density dependence plays at the scale of our study. Additional data may increase the differentiability of the other collinear variables in the future.

Our analysis suggests that immediate steps to mitigate the large declines in milkweed due to the use of herbicide-resistant crops in the breeding region is a key strategy for monarch restoration [12,14,78]. The Conservation Reserve Program (CRP) offers one particularly important mechanism for providing high-quality habitat for milkweed and the monarch butterflies reliant on them [78]; encouraging farmers and other land managers to include forbs and milkweed in seed mixes for CRP and other farm conservation incentive programmes could increase monarch habitat. Zalucki and Lammers [39] suggested a strategy for monarch conservation in the vast agricultural areas of the Corn Belt should be to increase milkweed abundance in the ‘matrix’, the land in between the remaining monarch habitat patches. This strategy could include focusing on roadsides and other right-of-way lands, yards and fence-rows [50,78]. In the southern USA, there has not been a single factor associated with extensive habitat loss; instead, multiple years of below-average precipitation may have had a larger effect on monarchs than habitat loss in this region. Improving monarch habitat across the species annual cycle may be more challenging, but doing so may ameliorate climate-related losses in any single step of the annual cycle.

Given that ≥92% of corn and soy agriculture in the northern USA is now glyphosate-tolerant [79], we might expect relatively little additional loss of agricultural fields as habitat for monarch butterflies [22]. Essentially, large declines in the carrying capacity of the eastern monarch population are nearly complete and predictions from both the partial least-squares regression and the best reduced-variable linear model suggest a roughly stationary population for the years 2012–2014 (figure 4). Thus, the ability to detect changes in monarch butterfly abundance in response to further increases in glyphosate application will probably be limited [22]. In the future, we may expect major drivers of population size to be associated with changes in grassland, including CRP land, and forest in the overwintering location. If threats to these habitats are mitigated, then we would expect climate-induced stochastic variation of the eastern migratory population of monarch butterfly around a relatively stationary population size (electronic supplementary material, appendix D).

## 5. Conclusion

We began our threats assessment with three times as many variables as years in our time series of monarch butterfly population size. Partial least-squares regression focused our attention on a subset of the 76 variables, leading to a single best model comprised of three covariates (i.e. glyphosate use, number of warm days in early June, minimum August temperature) explaining greater than 90% of the annual variation in abundance. A structural equation model implicates the loss of milkweed as the mechanism by which glyphosate application influences monarch butterfly population size. To offset this loss of milkweed, we suggest that a strategy of restoring habitat in a variety of areas including CRP lands, public and private lands, roadsides and marginal agricultural areas, as well as protecting habitat where monarchs overwinter, would help increase monarch populations [78] and thereby reduce the probability of extirpation. Further, we suggest that these conservation efforts should proceed quickly to avoid further monarch butterfly population declines.

## Supplementary Material

Appendix A

## Supplementary Material

Appendix B

## Supplementary Material

Appendix C

## Supplementary Material

Appendix D

## Data accessibility

The data are available via ScienceBase (http://dx.doi.org/10.5066/F7P55M8G) and the code is provided in electronic supplementary material, appendix B and via Bitbucket (https://my.usgs.gov/bitbucket/projects/UMESC/).
