# Supplementary material for: Monarch butterfly population decline in North America: identifying the threatening processes
Source: R Soc Open Sci. 2017 Sep 20;4(9):170760. doi: 10.1098/rsos.170760 (PMC5627118; doi:10.1098/rsos.170760)
Supplement: Appendix A [file rsos170760supp1.docx]

**Appendix A**. Graphical summaries of the predictor variables used in the analyses describing annual overwintering population size (ha).
