# Supplementary material for: Monarch butterfly population decline in North America: identifying the threatening processes
Source: R Soc Open Sci. 2017 Sep 20;4(9):170760. doi: 10.1098/rsos.170760 (PMC5627118; doi:10.1098/rsos.170760)
Supplement: Appendix C [file rsos170760supp3.docx]

**Appendix C**. Proportional change in the estimated slope for cumulative glyphosate application with the introduction of potential confounding variables.

The best reduced-variable model was comprised of cumulative glyphosate application and two climate variables (Table 2). A portion of the variation attributed to glyphosate application may be due to confounding variables also highly loading on component 1 of the partial least squares regression. We tested for confounding by introducing cumulative overwinter forest loss (Ramirez.cumul), previous year’s abundance, total neonicotinoid application (totalneon_NC), and total Dicamba application (Dicamba_S) into the best reduced-variable model and assessing change in the slope estimate of glyphosate application. The slope for glyphosate application changed <|6%| for Dicamba application, neonicotinoid application and previous year’s abundance (Figure C1). However, this slop estimate changed by ~25% in the presence of cumulative overwinter forest loss, indicating that some proportion of the variation attributed to glyphosate application may in fact be attributable to overwinter forest loss. When the obverse was considered, the magnitude of the slopes for the other variables changed considerably in the presence of glyphosate. For instance, the slope for total forest loss declined 72% and previous year's abundance declined 93% in the presence of glyphosate. Both neonicotinoids and dicamba application changed more than 100%, with their sign changing from negative to positive in the presence of glyphosate.

**
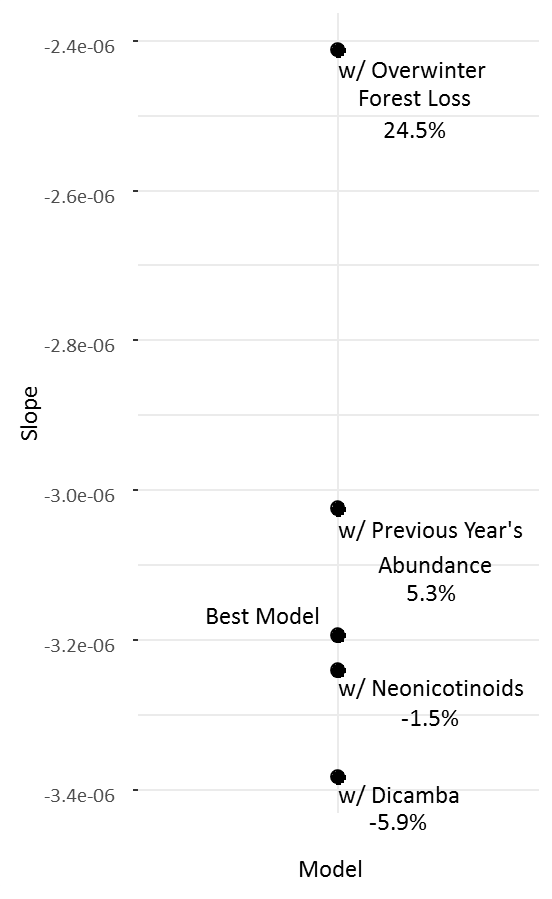
**

Figure C1. Slope estimate for glyphosate application (kg/ha) without (Best Model) and with potential confounding variables.
