# Supplementary material for: Monarch butterfly population decline in North America: identifying the threatening processes
Source: R Soc Open Sci. 2017 Sep 20;4(9):170760. doi: 10.1098/rsos.170760 (PMC5627118; doi:10.1098/rsos.170760)
Supplement: Appendix D [file rsos170760supp4.docx]

**Appendix D**. Stability analysis and sources of stochasticity in the time series of the eastern migratory population of monarch butterflies overwintering in Mexico

Semmens et al. (2016) estimated the process noise (standard deviation) in the overwintering population time series to be 0.49 (95% CI 0.28–0.80). This variability in population size drives the extinction probability for the species, especially when the population size is low. Semmens et al. (2016) attributed this high-process noise to stochastic environmental events such as extreme temperatures. We calculated the standard deviation of the residuals of equation 1 after attributing the deterministic portion to cumulative glyphosate application. The standard deviation of the time series after removal of the trend determined by glyphosate application was 0.25, approximately half of the process noise estimated by Semmens et al. (2016). The two climate covariates in the best reduced-variable regression, number of days with >21°C temperatures in Period 1 (sd = 0.41) and minimum temperature in August (sd = 0.68), each exhibited variation on the order of that reported by Semmens et al. (2016) and 50–100% more than the residual variation.

Stability analysis.— Increasing variability with time has been associated with population extirpation (Drake and Griffen 2010, Clements and Ozgul 2016) and community-level regime shifts (Carpenter et al. 2011). To this end, we calculated eight early warning signals using a moving window of 50% of the residuals of the time series after attributing the trend to glyphosate application (Dakos et al. 2012); these early warning signals were 1) the autoregressive coefficient at lag-1, 2) the inverse of this coefficient, which is the return rate, 3) density ratio (ratio of low frequencies to high frequencies), 4) autocorrelation, 5) standard deviation (2^nd^ moment), 6) coefficient of variation, 7) skewness (3^rd^ moment) and 8) kurtosis (4^th^ moment). Each of these metrics characterize different aspects of the statistical nature of the residuals of the original time series, after accounting for glyphosate application. For instance, the lag-1 autocorrelation describes the slowness of recovery from natural perturbations (van Nes and Sheffer 2007); variance increases with the accumulating impact of non-decaying shocks (Carpenter and Brock 2006). We calculated these early warning signals with the *earlywarnings* package in R (Dakos and Lahti 2013).

Following Dakos et al. (2008), we used Kendall’s τ to assess trends in the leading indicators. We should expect each of these metrics aside from return rate to increase in time if the population is approaching a critical transition such as (quasi-)extinction or, conversely, decrease in time if the population is stabilizing around a new stationary population size. Each of the metrics supports the supposition of the population stabilizing around a new stationary population size (Figure D1). Autocorrelation at lag-1 (Kendall τ = -0.212), skewness (Kendall τ = -0.727), and kurtosis (Kendall τ = -0.788) all decreased in time. Return rate (Kendall τ = 0.394) increased in time. Caution is warranted, however, because the sample size (i.e., number of time steps) associated with this time series is small, which could lead to instability and, hence, reduced reliability of these metrics as early warnings (Bœttiger and Hastings 2012). Thus, the prudent approach may be to update these analyses annually to evaluate changes in population status that may be forthcoming with changes in these metrics.

Figure D1. Pattern in eight early warning signals for the time series (exponentiated) of the eastern migratory population of monarch butterflies overwintering in central Mexico.

Sources of stochastic variation.— There are three years within the time series of overwinter population size having an outsized influence on the best model, 1996 (year 4), 2003 (year 11), and 2013 (year 21) (Fig. D2). It is unclear what the underlying process is leading to these three relatively aberrant years. We can amend equation 1 to separate the stochastic component into small and large perturbations. Recall, *f(x,θ)* is the deterministic component describing long-term changes in abundance whereas *g(x,θ)dW* is comprised of perturbations occurring at each time step. If we amend equation 1 to read: $dx=f\left( x,\theta\right)dt+g\left( x,\theta\right)dW+dJ_{t}$, we now include *dJ_t_*, which is defined as a jump process depicting large, one-time shocks uncorrelated in time (Dakos et al. 2012). In this new formulation, *g(x,θ)dW* is defined to include only small perturbations leading to diffusion in the time series.


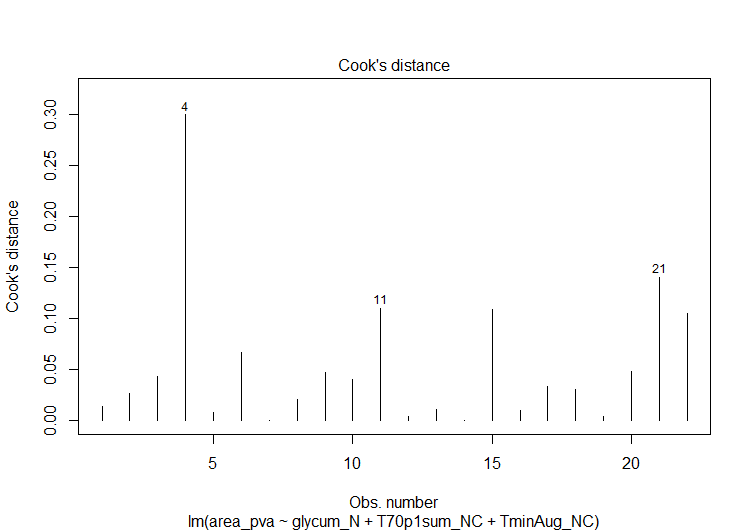


Figure D2. Cook’s distance measuring the effect of deleting a given year from the best model for describing the area occupied by monarch butterflies in winter in Mexico.

By fitting a non-parametric drift-diffusion-jump model to the time series of each of the climate variables in our best model, we find the jump intensity increases coincidentally in 1996 and 2003 (Fig. D3). We hypothesize that the outsized influence of 1996 is likely attributable to the number of >21°C days in Period 1 (early May), whereas the influence of 2003 is likely attributable to the minimum temperature in August. Why the time series of overwinter population size seems not to respond to the dramatic increase in jump intensity in 2011 for August minimum temperature is unknown, but may be a case where other factors occurring earlier in the annual cycle preclude species response.

The aberrancy of 2013 (year 21) is unclear as well; other climate variables identified as strongly loading in the partial least squares regression do not show correspondence in the manner that the number of >21°C days in Period 1 (early June) and minimum temperature in August do.


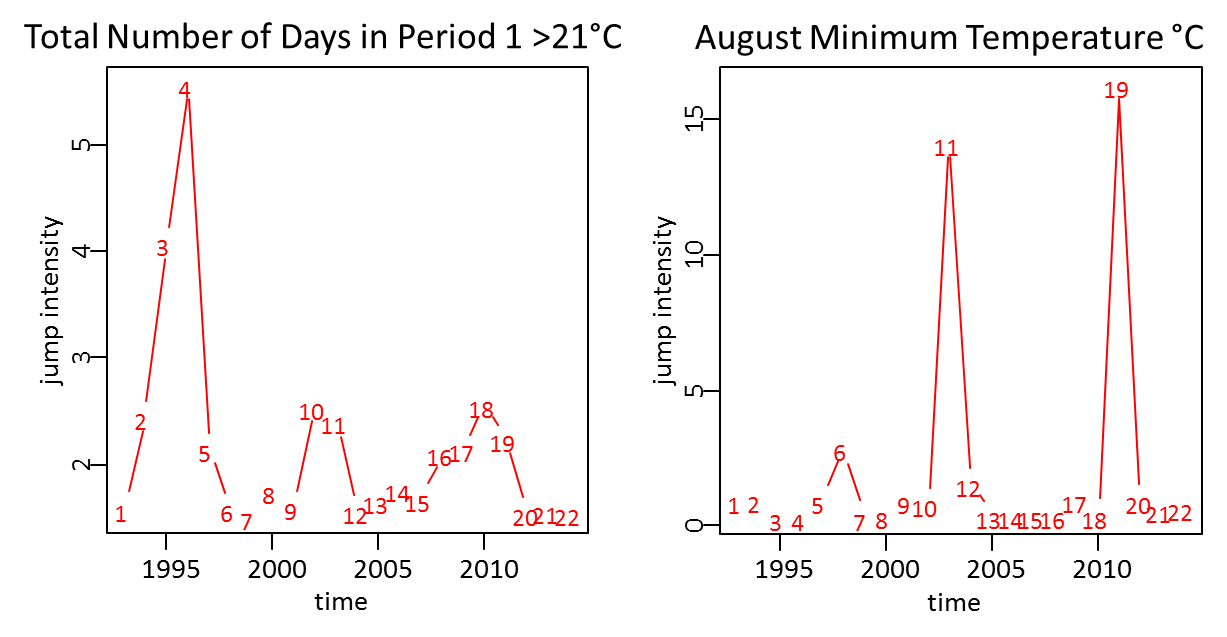


Figure D3. Annual jump intensity (dJ_t_) for the two time series of climate variables included in the best model describing the area occupied by monarch butterflies in winter in Mexico.

Literature Cited

Bœttiger, C. and Hastings, A. Quantifying limits to detection of early warning for critical transitions. Journal of the Royal Society Interface **9**, 2527–2539 (2012).

Carpenter, S. R. and Brock, W. A. Rising variance: a leading indicator of ecological

transition. Ecol. Lett. **9**, 308–315 (2006).

Carpenter, S. R., Cole, J. J., Pace, M. L., Batt, R., Brock, W. A., et al. Early warnings of regime shifts: a whole-ecosystem experiment. Science **332**, 1079–1082 (2011).

Dakos, V., Carpenter, S. R., Brock, W. A., Ellison, A. M., Guttal, V., et al. Methods for detecting early warnings of critical transitions in time series illustrated using simulated ecological data. PLoS One **7(7)**, e41010 (2012).

Dakos, V., and Lahti, L. R Early Warning Signals Toolbox. The R Project for Statistical Computing, <http://cran.r-project.org/web/packages/earlywarnings/index.html> (2013).

Dakos, V., Scheffer, M., van Nes, E. H., Brovkin, V., Petoukhov, V., and Held, H. Slowing down as an early warning signal for abrupt climate change. Proceedings of the National Academy of Sciences of the USA **105**, 14308–14312 (2008).

Drake, J., and Griffen, B. Early warning signals of extinction in deteriorating environments. Nature **467**, 456–459 (2010).

Semmens, B. X., Semmens, D. J., Thogmartin, W. E., Wiederholt, R., López-Hoffman, L., Diffendorfer, J. E., Pleasants, J., Oberhauser, K., and Taylor, O. Quasi-extinction risk and population targets for the Eastern, migratory population of monarch butterflies (*Danaus plexippus*). Scientific Reports **6**, 23265 (2016).

van Nes, E. H. and Scheffer, M. Slow recovery from perturbations as a generic indicator of a nearby catastrophic shift. American Naturalist **169**, 738–747 (2007).
